# Temporal lobe activation during episodic memory encoding following traumatic brain injury

**DOI:** 10.1038/s41598-021-97953-6

**Published:** 2021-09-22

**Authors:** Abbie S. Taing, Matthew E. Mundy, Jennie L. Ponsford, Gershon Spitz

**Affiliations:** 1 Turner Institute for Brain and Mental Health, School of Psychological Sciences, Monash University, Clayton, VIC 3800 Australia; 2 Monash Epworth Rehabilitation Research Centre, 185-187 Hoddle Street, Richmond, VIC 3121 Australia

**Keywords:** Neuroscience, Psychology

## Abstract

The temporal lobes are critical for encoding and retrieving episodic memories. The temporal lobes are preferentially disrupted following a traumatic brain injury (TBI), likely contributing to the difficulties observed in episodic memory. However, the underlying neural changes that precipitate or maintain these difficulties in individuals with TBI remains poorly understood. Here, we use functional magnetic resonance imaging (fMRI) to interrogate the relationship between temporal lobe activation and encoding of episodic stimuli. Participants encoded face, scene, and animal stimuli during an fMRI run. In an out-of-scanner task, participants were required to correctly identify previously displayed stimuli over two presentation runs (each in-scanner stimuli presented twice). Forty-three patients with moderate-severe TBI were recruited and compared with 38 demographically similar healthy controls. The pattern of behavioural performance between groups depended on the stimuli presentation run. The TBI group demonstrated poorer episodic memory for faces and scenes during the first presentation, but not the second presentation. When episodic memory was analysed across all presentation runs, behavioural deficits were only apparent for faces. Interestingly, processing of faces emerged as the only between group-difference on fMRI, whereby TBI participants had an increased signal in the middle temporal gyrus extending to the superior temporal sulcus. These findings provide evidence to suggest that following TBI: (a) episodic memory is preferentially impaired for complex stimuli such as faces, and (b) robust behavioural inefficiencies are reflected in increased activation in specific temporal lobe structures during encoding.

## Introduction

Traumatic brain injury (TBI) can cause focal and diffuse disruption to multiple brain systems. Pathology is most widely observed in frontal and temporal cortices^1^, and as such impairments in processing speed, attention, executive function, and memory are most common^2,3^. Invariably, all individuals with a moderate-severe TBI will experience an initial transient period of impaired consciousness with amnesia and general confusion known as post-traumatic amnesia (PTA)^4^. Although most individuals emerge from PTA, many will experience ongoing difficulty with episodic memories^5,6^. Episodic memories involve the ability to learn, store, and retrieve information about personal experiences^7,8^. Deficits in episodic memory can interfere with crucial skills such as new learning and task completion, and therefore can significantly limit functional independence and productivity^9^.

Neuroanatomically, episodic memory is supported by a brain network spanning several regions in the frontal and parietal cortices^10,11^. However, structures contained within the temporal lobes are arguably the most crucial for particular aspects of episodic memory^7,12^. The temporal lobes house the hippocampus, and the entorhinal, perirhinal, and parahippocampal cortices—collectively known as the medial temporal lobes^13,14^. The hippocampus and other medial temporal structures are involved in the encoding of episodic stimuli, and modulation of activity in these regions has been found to predict subsequent recollection of the encoded stimuli^15^.

The temporal lobes are preferentially affected following TBI^1^. The location of the temporal lobes and their proximity to the middle cranial fossa makes them highly susceptible to acceleration-deceleration forces present during injury^16,17^. Damage to white matter tracts projecting into and out of the temporal lobes (e.g. fornix) due to diffuse axonal injury is also common^18^. Structures within the temporal lobes, such as the hippocampus and other limbic structures, are particularly susceptible to hypoxic insults and excitotoxicity effects^12^. As such, atrophy in the hippocampus and fornix is common after injury^19^, and has been associated with impaired memory^20–22^.

Functional neuroimaging studies in the TBI population have also implicated the temporal lobes in impaired episodic memory. Individuals with TBI tend to display increased activity in various regions, including the temporal lobes, during encoding of episodic stimuli when compared with healthy controls^23–26^. One limitation of these previous studies is that most have failed to use paradigms that may reflect the type of episodic memory deficits that individuals with TBI may experience in their daily life. For example, some studies used abstract visual stimuli^23,24,26^ and/or have only assessed short-term recognition^23,24^. Gillis and Hampstead addressed these methodological shortcomings by using realistic images of common objects and assessed long-term retention outside of the scanner^25^. However, this study had a relatively small sample size (n = 7) and no significant difference in behaviour was observed between the TBI group and healthy controls. Here, we rectify this gap by using a larger sample to investigate the extent to which episodic memory is impaired following TBI.

We also extend upon past studies by examining whether deficits occur for common categories of everyday stimuli. We focus specifically on the temporal lobes given their role in processing of various categories of stimuli^14^. For example, processing of common stimuli such as faces, scenes, and animals has been found to recruit specialised temporal lobe structures^27,28^. Exposure to faces robustly activates regions in the middle fusiform gyrus (‘fusiform face area’), lateral inferior occipital gyrus (‘occipital face area’), and superior temporal sulcus^29–31^; exposure to scenes activates the posterior parahippocampus ('parahippocampal place area')^32^; and exposure to animals activates the bilateral fusiform gyrus^27,33^. Although some degree of impairment is expected given the high prevalence of temporal pathology and specialised processing of various stimuli in this area, it is possible that not all stimuli are equally impaired following injury. Past studies of amnestic patients have demonstrated stimulus-sensitive impairments for stimuli such as faces and scenes^34,35^. In the TBI population, impairment of face recognition has also been previously documented^36^. Processing of faces may be particularly impaired because these complex stimuli are processed differently than other visual stimuli^37^. For example, faces are unique in that they are processed in a holistic and configural manner^38,39^ and multiple cortical areas are required to process different aspects of face perception (e.g. identification, expression)^38,40^.

The temporal lobes play a critical role in episodic memory, which is frequently impaired following TBI. Therefore, the present study specifically focusses on the temporal lobes to examine how episodic memory impairments may affect aspects of everyday memory. We used an fMRI task to measure temporal lobe processing during encoding of faces, scenes, and animals. Recognition memory was subsequently probed in an out-of-scanner behavioural task. In line with previous studies^23–26^, we hypothesised that the TBI group would display greater temporal lobe activation during stimulus encoding compared to healthy controls. Furthermore, we hypothesised that greater activity during encoding would be associated with poorer recognition memory. Lastly, we hypothesised that individuals with TBI would be most impaired for complex stimuli such as faces and scenes.

## Materials and methods

### Participants

**Figure 1:**
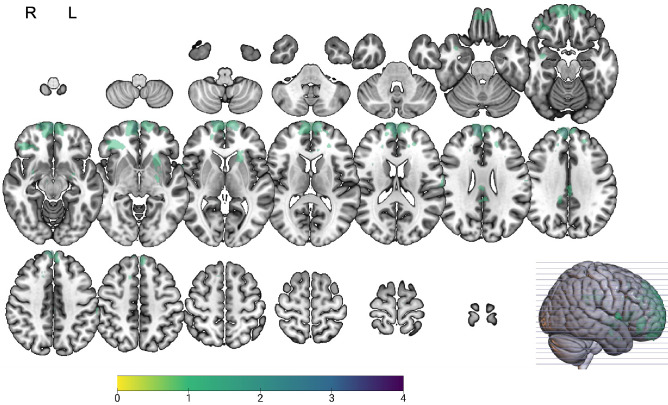
Lesion overlay plot of all TBI participants. Maps were overlaid on a T1 template in MNI space. Purple colour indicates greater lesion overlap across participants.

Forty-three participants (31 males, 12 females) who had sustained moderate-severe TBI, determined prospectively using the Westmead Post Traumatic Amnesia Scale (WPTAS) ^41^, were recruited from Epworth Healthcare, either from successive admissions to the inpatient ward or via a longitudinal follow-up database (Table 1 and Supplementary Table S1). TBI participants were recruited at an average of 11 months post-injury (*SD* = 11.57 months, range 0.69–34.82 months) and were scanned at three different sites. TBI participants predominantly had prefrontal, temporal, and subcortical pathology (Fig. 1; see also Supplementary Table S1 for imaging findings). Exclusion criteria included age < 18 or > 75 years, prior history of TBI or other neurological conditions, significant psychiatric or substance abuse history, and MRI contraindication. Thirty-eight healthy controls (26 males, 12 females) of similar age, sex, and education were also recruited (Table 1). There were no significant group differences on any of the demographic variables (*P* > 0.05). Six TBI participants and nine healthy controls were excluded due to a technical error resulting in poor coverage of the temporal lobes during data collection. Additionally, one healthy control participant was excluded due to excessive movement in the scanner. Despite this, it should be noted that our fMRI sample size is larger than previous neuroimaging studies^23–25^ of episodic memory in the TBI population. Written informed consent was provided by all participants in accordance with the Declaration of Helsinki. This study was performed in accordance with the relevant guidelines and regulations approved by Monash Health/University Human Research Ethics Committee.

**Table 1:** Demographic information and clinical characteristics of the TBI and healthy groups.

Demographic variables	Traumatic brain injury, Mean (SD)	Healthy controls, Mean (SD)
Age (years)	40.77 (16.46)	40.05 (17.14)
Sex (male/female)	31/12	26/12
Education (years)	14.23 (2.94)	14.68 (2.79)
Time since injury (months)	11.07 (11.57)	–
PTA (days)	26.88 (28.06)	–
GCS (lowest)	9.19 (4.23)	–

*GSC* Glasgow Coma Scale, *PTA* post-traumatic amnesia.

PTA duration were available for *n* = 41 patients; GCS were available for *n* = 42 patients.

### Episodic memory paradigm

**Figure 2:**
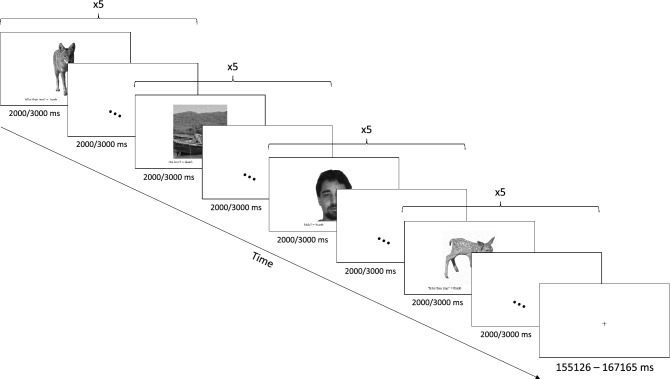
Schematic diagram of the episodic encoding task. Participants were presented with 20 images per block (with five images from each stimulus category presented consecutively) and were instructed to respond to the various stimuli on screen based on set criteria. Each stimulus was presented for 2/3 s, followed by an inter-stimulus duration of 2/3 s. Stimuli were sourced from the Psychological Collection of Images at Stirling database (available at pics.stir.ac.uk), the Centre for Vital Longevity Face Database (available at agingmind.utdallas.edu/facedb), as well as other publicly available free-to-use image databases.

The episodic memory paradigm is a task adapted from Mundy et al. to probe episodic memory encoding and recognition (Fig. 2)^35,42^. Participants were presented images of faces, scenes, and animals while in the scanner. They were instructed to respond, using trigger buttons, to each stimulus based on set criteria to ensure attention was maintained throughout the task (e.g. decide whether the face is male or female; whether a scene looks hot or cold; whether an animal is shorter or taller than a human man). The fMRI task consisted of six blocks, each block consisted of 20 images from the three stimulus categories. There were a total of 60 unique stimuli, each presented twice throughout the session. To ensure equivalent task difficulty, stimulus presentation was reduced for individuals recruited further along post-injury. Each stimulus was presented for 3 s and followed by a 3 s inter-stimulus interval for those less than 1 year post-injury; or stimulus presentation of 2 s and followed by a 2 s inter-stimulus interval for those more than 1 year post injury. Five rest blocks were presented after the 1st–5th experimental blocks.

Memory of the stimuli presented in the fMRI task was probed in an out-of-scanner task. Recognition of the episodic stimuli was assessed by instructing participants to classify stimuli as “old” (i.e. images seen during the fMRI task) or “new” (i.e. images that were not seen during the fMRI task). Participants rated a total of 60 old and 60 new stimuli, each presented twice (i.e. 240 stimuli in total). Participants rated all old and new stimuli, before having to rate each stimulus again. Thus, recognition memory was assessed over two presentation runs. To assess recognition ‘confidence’, images were rated on a scale from “definitely old” to “definitely new”. Responses were considered correct/incorrect regardless of confidence level (secondary analysis indicated no significant differences in confidence rating between groups—see “Confidence rating analysis” in Supplementary for further detail). Performance for this task was assessed using accuracy and reaction times.

### MRI acquisition

Structural and functional MR images were acquired across three scanners, using 3.0 Tesla Siemens Magnetom Skyra scanners and 20/32-channel head coils. Site 1 and 2—functional images were acquired using single-shot gradient-echo planar imaging (EPI) with the following parameters: repetition time (TR) = 2.75 s; echo time (TE) = 30 ms; flip angle = 90°; 220 × 220 matrix; voxel size = 3.4 × 3.4 × 3.0 mm. A high-resolution 3D T1-weighted image covering the entire brain was also acquired for anatomical reference (TR = 2.3 s; TE = 2.32 ms; flip angle = 8°; 236 × 350 matrix; voxel size = 0.9 × 0.9 × 0.9 mm). Site 3—functional images were acquired using single-shot EPI with the following parameters: TR = 0.74 s; TE = 39 ms; simultaneous multi-slice (SMS) acceleration factor = 8; flip angle = 52°; 210 × 210 matrix; voxel size = 2.4 × 2.4 × 2.4 mm. A single-band reference scan was also obtained for EPI registration purposes with the following parameters: TR = 6.37 s; TE = 39 ms; flip angle = 52°; 210 × 210 matrix; voxel size = 2.4 × 2.4 × 2.4 mm. A high-resolution 3D T1-weighted image covering the entire brain was also acquired with the following parameters: TR = 2.0 s; TE = 2.03 ms; flip angle = 8°; 256 × 256 matrix; voxel size = 1.0 × 1.0 × 1.0 mm. Due to reduced brain coverage (due to using clinical scanners), and a-priori hypotheses, we focused on the temporal lobes.

### Statistical analysis

#### Behavioural and demographic data

Behavioural and demographic data were analysed using R version 3.6.0 (R Core Team, 2019). Two-tailed independent samples t-tests were used to examine group differences on the demographic variables (i.e. age, sex, and years of education). Behavioural data were screened for normality, transformed (if necessary), and assessed for violation of statistical assumptions prior to analysis. Outcome measures were analysed using linear mixed models to account for clustering or non-independence of measures within participants. Task performance was assessed using dprime and reaction time. Dprime measured task accuracy, accounting for the signal (hits) and noise (false alarms). Reaction times were generated by obtaining the average reaction time per stimulus category. Accuracy was assessed by modelling stimulus category, group, and an interaction (stimulus category × group) as fixed effects, and participant as a random effect. For reaction time, the data were inversely transformed, and a model was fitted with stimulus category, group, and an interaction (stimulus category × group) as fixed effects, and participant as a random effect. Age and education were also added as covariates in the models given their influence on memory performance^43,44^ and reaction time^45^. Results are reported for overall performance (i.e. first and second presentations combined) as well as separately for each presentation run. Given the expected lower performance for the TBI group compared to healthy controls, where appropriate post-hoc analyses were conducted using one-tailed t-tests, corrected for multiple comparison.

### Imaging data

#### MRI preprocessing

Prior to preprocessing, lesions were manually segmented using MRIcron (http://www.mricro.com/mricron). Preprocessing was performed using fMRIPrep 20.0.0 (Esteban et al., 2018) and involved the application of the following step: undistortion of EPI data, realignment, normalisation, and estimation of confounds. Further information about the MRI preprocessing can be accessed in the Supplementary (see “Detailed MRI preprocessing”).

#### fMRI analysis

fMRI data were analysed using FSL’s FEAT version 6.0.2 (FMRIB's Software Library, www.fmrib.ox.ac.uk/fsl). In the first level analysis, contrasts between each stimulus category (i.e. faces, scenes, animals) and the rest blocks were generated for each participant. The onset times for each contrast corresponded to the first stimulus presentation of each category. To reduce motion-related artifacts, additional regressors using a modified method of the anatomical CompCor that explained 50% of the variance were also included in the first level model^46^. Differences in brain activation were assessed in a 2 (group: TBI vs. healthy controls) × 3 (stimulus category: faces, scenes, and animals) factorial design using FLAME 1 + 2 mixed effects with automatic outlier de-weighting. Scanner site was included as a covariate. Given our hypothesis regarding structures in the temporal lobes and their role in learning and processing of category-specific stimuli, an a-priori region of interest (ROI) mask of the temporal lobes was generated using the MNI Structural Atlas (Supplementary Fig. S1) and used in the group level analysis. Imaging findings are reported using a cluster level threshold of *Z* > 3.1 and a family wise error cluster correction threshold of *P* < 0.05. FEAT contrast of parameter estimates (COPE) were extracted from significant clusters at the group level. To investigate differences in BOLD response, two-tailed independent samples t-tests were conducted using COPE values. Finally, association between BOLD response and behavioural performance on the episodic recognition task were assessed using Pearson correlations.

## Results

### Episodic memory following TBI is selectively impaired for face stimuli

**Figure 3:**
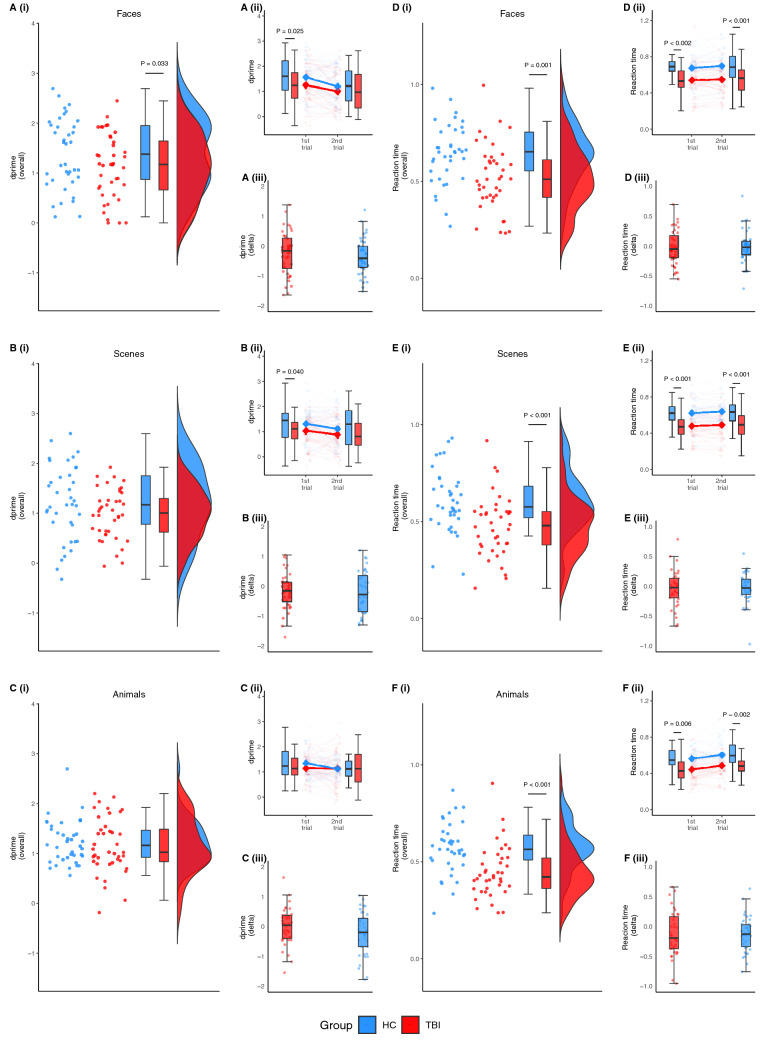
Behavioural results for the episodic recognition task. Plots show individual datapoints along with boxplots and/or violin plots showing distribution. Panel left—plots of accuracy according to stimulus category, as measured using dprime (higher values denote better performance). The TBI group had significantly poorer accuracy than healthy controls when retrieving faces overall (*P* = 0.033; **Ai**) and for the first presentation run (*P* = 0.025; **Aii**). No significant differences in accuracy were apparent between groups for faces on the second trial (*P* > 0.05; **Aii**) nor was the change in accuracy between presentation runs (*P* > 0.05; **Aiii**). There was a trend for the TBI group to have lower accuracy than healthy controls for overall scenes performance, although this did not reach statistical significance (*P* = 0.059; **Bi**). However, the TBI group had poorer accuracy for scenes when considering performance on the first presentation run (*P* = 0.040; **Bii**). No significant difference in accuracy was apparent between groups for scenes on the second presentation run (*P* > 0.05; **Bii**) nor was the change in accuracy between presentation runs (*P* > 0.05; **Biii**). There were no significant differences in accuracy between groups for animals overall (*P* > 0.05; **Ci**), nor on the first presentation run (*P* > 0.05; **Cii**) or second presentation run (*P* > 0.05; **Cii**). There was also no significant difference in the change in accuracy between presentation runs for animals (*P* > 0.05; **Ciii**). Panel right—plots of reaction times according to stimulus category (note: reaction time was inversely transformed; higher values denote faster performance). As expected, the TBI group was slower than healthy controls when responding to faces (**D**), scenes (**E**), and animals (**F**) overall as well as for the first and second presentation runs (*P* < 0.05). Reported *P*-values were adjusted for multiple comparisons.

First, we investigated whether recognition of episodic memories was impaired following TBI. To do this, we performed a linear mixed model assessing memory recognition accuracy (old vs. new, as measured using dprime) on the episodic recognition task. Overall, the TBI group demonstrated significantly poorer recognition accuracy than healthy controls (95% CI, − 0.52 to − 0.01; *P* = 0.030). Post-hoc analyses indicated that the group difference was driven by a significant difference in accuracy for faces (95% CI, − 0.53 to 0.02; *P* = 0.033; Fig. 3Ai). There was a trend whereby the TBI group had lower accuracy than healthy controls for scenes, however, this did not reach statistical significance (95% CI, − 0.49 to 0.06; *P* = 0.059; Fig. 3Bi). There was no significant difference between the groups in accuracy for animals (95% CI, − 0.33 to 0.22; *P* = 0.338; Fig. 3Ci).

### Episodic memory disruption in TBI is only evident during the first stimuli presentation

We examined whether episodic memory differed between the first and second presentation runs. During the first presentation run, the TBI group demonstrated poorer accuracy than healthy controls, regardless of stimulus type (95% CI, − 0.60 to − 0.01; *P* = 0.022). Post-hoc analyses indicated worse accuracy for the TBI group compared to healthy controls for faces (95% CI, − 0.61 to − 0.001; *P* = 0.025; Fig. 3Aii) and scenes (95% CI, − 0.57 to 0.03; *P* = 0.040; Fig. 3Bii). There was also a trend for lower accuracy for animals, although this did not reach statistical significance (95% CI, − 0.50 to 0.11; *P* = 0.105; Fig. 3Cii). In contrast, there was no significant difference in accuracy between groups when considering performance from the second presentation run (95% CI, − 0.49 to 0.12; *P* = 0.121; Fig. 3Aii–Cii). To determine whether the change in performance across recognition trials differed between groups, the change in accuracy (dprime delta) was calculated by subtracting dprime scores for the first presentation run from the second presentation run. Overall, there was no significant difference between groups in the change in accuracy across presentation runs (95% CI, − 0.17 to 0.41; *P* = 0.212; Fig. 3Aiii–3Ciii).

### Reaction times are quickest for face stimuli

Next, we investigated whether reaction time was significantly different between the groups and whether it varied according to the type of stimulus. Overall, reaction time was greater for individuals with TBI compared to healthy controls, irrespective of stimulus type (95% CI, − 0.19 to − 0.06; *P* < 0.001). Post-hoc analyses revealed that the TBI group was significantly slower than healthy controls in responding to faces (95% CI, − 0.19 to − 0.06; *P* < 0.001; Fig. 3Di), scenes (95% CI, − 0.20 to − 0.06; *P* < 0.001; Fig. 3Ei), and animals (95% CI, − 0.19 to − 0.06; *P* < 0.001; Fig. 3Fi). Across both groups, reaction time also varied depending on the stimulus category: the TBI group was quicker to respond to faces than scenes (95% CI, 0.01 to 0.08; *P* = 0.004) and animals (95% CI, 0.04 to 0.11; *P* < 0.001); similarly, healthy controls were quicker to respond to faces than scenes (95% CI, 0.003 to 0.07; *P* = 0.014) and animals (95% CI, 0.04 to 0.11; *P* < 0.001).

Similar patterns of findings were observed when reaction times were examined for the first and second presentation runs. The TBI group was slower than healthy controls, irrespective of stimulus type, in both the first (95% CI, − 0.20 to − 0.07; *P* < 0.001) and second presentation runs (95% CI, − 0.22 to − 0.08; *P* < 0.001). Post-hoc analyses revealed that the TBI group was slower than healthy controls in both the first and second presentation runs in responding to faces, scenes, and animals (*P* < 0.05; Fig. 3Dii–Fii). Across both groups, reaction times also varied depending on the stimulus category. In both the first and second presentation runs, the TBI group was quicker to respond to faces than scenes and animals (*P* < 0.05); similarly, healthy controls were quicker to respond to faces than scenes and animals, and animals than scenes (*P* < 0.05). To determine whether the change in reaction times across presentation runs differed between groups, the change in reaction times (reaction time delta) was calculated by subtracting reaction times for the first presentation run from the second presentation run. There was no significant difference between the groups in the change in reaction times across presentation runs (95% CI, − 0.06 to 0.03; *P* = 0.431; Fig. 3Diii–Fiii).

Given this pattern of results, we further investigated whether poorer performance for faces in the TBI group was driven by a speed-accuracy trade-off. To do this, we included reaction time as a covariate in the linear mixed model. Results indicated that participants with TBI still performed significantly poorer than healthy controls (95% CI, − 0.64 to − 0.03; *P* = 0.015), suggesting that this pattern of performance was not due solely to a speed-accuracy trade-off.

### fMRI task activates the stereotypical regions underpinning encoding of episodic stimuli

**Figure 4:**
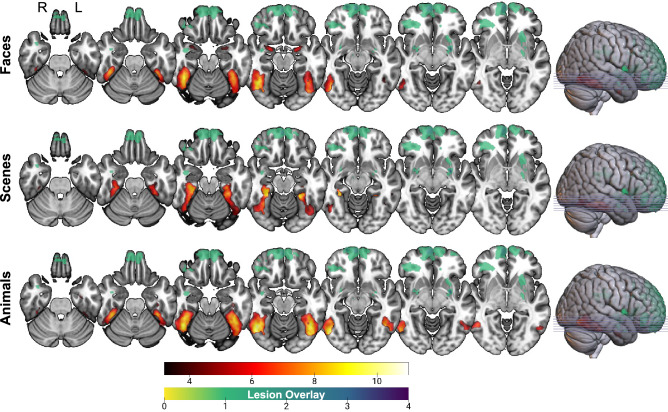
Overall functional activity elicited during the episodic encoding task for the whole sample. Significant clusters during encoding of faces, scenes, and animals are shown in red/yellow. There was no overlap between these clusters and TBI lesions (green).

To demonstrate that our task elicited activations in stereotypical areas involved with the canonical network that support encoding of episodic stimuli, we first included all participants in an analysis looking at the average activation for faces, scenes, and animals (Fig. 4; see also Supplementary Tables S2 for peak clusters). During encoding of face stimuli, significant clusters were noted in face-selective areas including the bilateral fusiform gyrus^29–31^ and amgydala^47^. During encoding of scene stimuli, significant clusters were noted in the bilateral fusiform gyrus extending to the parahippocampal gyrus^32^. Finally, during encoding of animal stimuli, significant clusters were noted in animal-selective area of the bilateral fusiform gyrus^27,33^.

### TBI patients show increased left middle temporal gyrus extending to the superior temporal sulcus activation during face processing

**Figure 5:**
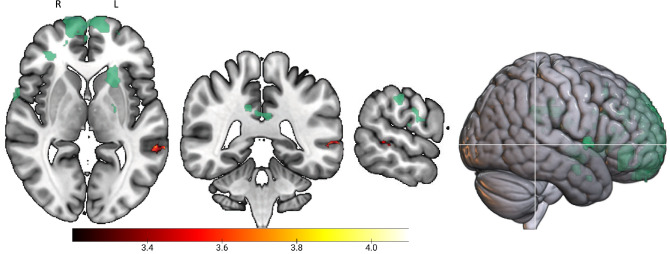
An increased activation in the left middle temporal gyrus extending to the superior temporal sulcus during encoding of faces was apparent for TBI compared to healthy controls. There was no overlap between this cluster (red/yellow) and TBI lesions (green).

Consistent with the behavioural results, group differences on imaging were apparent during encoding of faces. TBI participants showed increased activation in the left middle temporal gyrus extending to the superior temporal sulcus compared to healthy controls (Fig. 5; see also Supplementary Tables S3 for peak clusters).

In contrast, no significant group differences were apparent during encoding of scenes or animals.

### Activation in temporal structures during face encoding does not directly predict performance

To further explore the brain-behaviour relationship, FEAT analysis COPE values were extracted for the significant face cluster. We first investigated whether there was a significant difference in BOLD response between groups using an independent samples t-test (two-tailed). As expected, TBI patients displayed higher COPE values (*M* = 7.20, *SD* = 21.75) compared to healthy controls (*M* = − 9.86, *SD* = 23.16), *t*(18) = 3.05, *P* = 0.003 (Supplementary Fig. 2A). We further examined whether there was an association with behavioural performance on the episodic recognition task using Pearson correlations. Overall, there was no direct relationship between COPE values and the overall dprime scores for face stimuli, *r*(63) = − 0.17, *P* = 0.167 (Supplementary Fig. 2B). There was a weak negative correlation between COPE values and face dprime scores that approached significance when considering the first presentation run, *r*(63) = − 0.21, *P* = 0.088 (Supplementary Fig. 2C); there was no significant correlations between COPE values and face dprime scores for the second presentation run, *r*(63) = − 0.080, *P* = 0.527 (Supplementary Fig. 2D).

## Discussion

The present study focused on determining the role of temporal lobe activity in episodic memory following TBI. We showed for the first time, using converging evidence from behavioural and fMRI data, that episodic memory impairment following TBI appeared to be category-specific and related to a specific region within the temporal lobe. The TBI group demonstrated poorer recognition accuracy for faces and scenes, but only during the first re-exposure of these stimuli. When episodic memory performance was combined across presentation runs, impaired performance was only present for face stimuli. This particular deficit in face recognition manifested as an increased activation in the middle temporal gyrus extending to the superior temporal sulcus during face encoding. Overall, these findings suggest that following TBI: (a) episodic memory is preferentially impaired for complex stimuli such as faces, and (b) robust behavioural inefficiencies are reflected in increased activation in specific temporal lobe structures during encoding.

Broadly speaking, our findings are similar to those of previous studies of amnestic patients who show stimulus-sensitive impairments for complex stimuli such as faces and scenes^34,35^. Our findings are also in concordance with a previous study demonstrating impaired face recognition in the TBI population^36^. Valentine et al. exposed participants to a range of facial recognition and learning tasks and found that while performance varied, deficits were more apparent for tasks with greater demands^36^. More specifically, the most sensitive tasks were those which contained a larger number of faces to be encoded or had fewer presentations of the stimuli. Our task was comparably difficult in that participants were presented with a similar number of face stimuli which were only shown twice during the encoding phase; thus, it was not surprising we obtained a similar finding.

As expected, we found that the TBI group was generally slower than healthy controls in their reaction times. Both groups, however, responded more quickly to faces than to animals and scenes. This result somewhat aligns with a study conducted by Keightley et al. who found that participants reacted quicker to faces than scenes^48^. We further explored whether speed-accuracy trade off could account for our findings, given participants displayed poorer accuracy for face stimuli. We found that including reaction time as a covariate when determining between-group differences in face accuracy did not change the result. Instead, the rapid response to faces suggests that individuals with TBI may have performed superficial encoding of face stimuli, thus negatively impacting decision-making during recognition.

A unique aspect of our study was the inclusion of various categories of stimuli which allowed us to investigate whether there are selective impairments for specific categories. Interestingly, our results indicated that the TBI group found faces most difficult relative to healthy controls, as behavioural deficit was only apparent for these stimuli when overall performance was considered. One potential reason for these results is that faces are processed differently than other visual stimuli^37^. Indeed, facial processing is a complex phenomenon requiring multifaceted processes across widespread cortical areas^40^. In addition, unlike most other visual stimuli they are processed in a holistic and configural manner^38,39^; thus, discrimination requires attention to detail and subtle perception of variable facial features^40^.

An unexpected finding was that performance differed between the groups depending on the presentation run during the episodic recognition task. That is, the TBI group demonstrated impaired memory recognition compared to healthy controls during the first presentation run for faces and scenes, although no difference in performance was observed by the second presentation run. An explanation for this finding pertains to the fact that exposure to stimuli in the first presentation run may have led to more errors in the second presentation run because of source memory confusion (i.e. misattribution of contextual memory surrounding the information)^49,50^ and/or retroactive interference (i.e. new information detrimentally effects previously learnt information)^51^. This is relevant for “new” stimuli as exposure to these stimuli during the first presentation run may have resulted in an incorrect judgement of the stimuli as “old” (i.e. false positive) in the second presentation run. Consistent with this hypothesis is the fact that performance for both the TBI group and healthy controls was poorer for the second presentation run. However, the pattern of results suggest that healthy controls may have been more susceptible to these effects, perhaps due to ceiling effect. That is, there may be a higher probability of ‘regression to the mean’ for healthy controls in comparison to the TBI group. For these reasons, we suggest that performance on the first presentation run may be a better marker of episodic memory.

Although performance on the first presentation run showed that the TBI group was impaired for faces and scenes, processing of faces emerged as the only between group-difference on fMRI. Thus, fMRI finding also supports the notion of a specific disruption for faces, over-and-above scenes and animals. During encoding of faces, the TBI group showed an increased response in the left middle temporal gyrus extending to the superior temporal sulcus. In healthy controls, the middle temporal gyrus has been implicated in processing of facial expressions^52,53^. Moreover, the superior temporal sulcus forms part of the core face network^30^ and is involved in processing of dynamic facial features, such as eye gaze direction and emotional expression^54–56^. Taken together with the behavioural results, increased activation in these temporal structures suggests that individuals with TBI used more contextual cues, albeit less efficiently, when processing faces.

However, examination of activation in the significant face cluster (i.e. middle temporal gyrus/superior temporal sulcus) and recognition memory for face stimuli failed to show a clear brain-behaviour relationship. That is, no significant correlation was apparent between BOLD activity and face recognition performance. One explanation as to why we did not observe such association is because of the contribution of other brain regions implicated in encoding of episodic stimuli. For example, the frontal lobes are involved in the organisational aspects of episodic encoding^11^, including strategy use, allocation of resources, and planning^57,58^. Thus, it is possible that temporal lobe activation alone is not sufficient to fully capture this brain-behaviour relationship. Further investigation of the interactions between temporal lobe and other brain regions may help clarify this.

There were some limitations in our study. Our episodic memory paradigm only allowed us to investigate functional activity during stimulus encoding. Therefore, we could not comment on how temporal lobe structures are neurally implicated during recognition or retrieval of episodic stimuli following TBI. This may be an avenue for exploration in future research. An important consideration that could affect the interpretation of our fMRI results is the presence of structural injuries. In terms of focal lesions, these do not appear to overlap with the significant cluster and thus could not explain for difference in BOLD activation between groups. Despite this, it should be noted that only focal lesions were considered in the current study. Given the high frequency of diffuse axonal injury^18^ and the impact on memory^21,22^, future studies may consider also using diffusion tensor imaging (DTI) to quantify microstructural injury to white matter tracts. Another consideration of the current study was the heterogeneity of TBI participants. For example, the inclusion of individuals at various injury timepoints makes it difficult to discern the effects of recovery phase on episodic memory. Nonetheless, it is reassuring that deficits were apparent despite the heterogeneity of our participants and that the most robust finding (i.e. deficit for faces) was apparent on both behavioural and functional imaging modalities.

Despite the limitations, our study has several important implications. From a clinical perspective, it is generally acknowledged that individuals have generalised episodic memory deficits after injury. Our findings provide evidence to the contrary and show that impairment is more apparent for complex visual stimuli such as faces and scenes. Therefore, complex visual stimuli may be more suited to assess for episodic memory deficits given the higher specificity to distinguish impaired performance relative to healthy controls. More practically, our findings help clarify strategies that are helpful following TBI. An obvious clinical translation is the need to provide strategies that promote deeper processing to better aid memory for complex stimuli.

In conclusion, we found evidence demonstrating that individuals with TBI show impairment of episodic memory for complex stimuli and that this was associated with functional changes. Behaviourally, the TBI group demonstrated poorer recognition accuracy for faces and scenes initially, although deficits were only apparent for faces when performance was considered overall. Neurally, the behavioural impairment for faces manifested as an increased activation in the middle temporal gyrus extending to the superior temporal sulcus during face encoding. Overall, we provide preliminary evidence demonstrating that following TBI: (a) episodic memory impairment is domain specific and more broadly dependent on the complexity of the stimuli, and (b) robust behavioural inefficiencies are reflected in increased activation in specific temporal lobe structures during encoding.

## Supplementary Information

Supplementary Information.

## Data Availability

All data supporting the findings of this study can be requested from the corresponding author.

## Abbreviations

COPE: Contrast of parameter estimates
DTI: diffusion tensor imaging
EPI: echo planar imaging
fMRI: Functional magnetic resonance imaging
GSC: Glasgow coma scale
PTA: Post traumatic amnesia
SMS: simultaneous multi-slice
TBI: Traumatic brain injury
TE: Echo time
TR: Repetition time
WPTAS: Westmead post traumatic amnesia scale
