# Supplementary material for: Temporal lobe activation during episodic memory encoding following traumatic brain injury
Source: Sci Rep. 2021 Sep 22;11:18830. doi: 10.1038/s41598-021-97953-6 (PMC8458357; doi:10.1038/s41598-021-97953-6)
Supplement: Supplementary file 1 — Supplementary Information. [file 41598_2021_97953_MOESM1_ESM.docx]

**Temporal lobe activation during episodic memory encoding following traumatic brain injury**

Abbie S. Taing^1,2^, Matthew E. Mundy^1^, Jennie L. Ponsford^1,2^, Gershon Spitz^1,2*^

^1^ Turner Institute for Brain and Mental Health, School of Psychological Sciences, Monash University, Clayton, Victoria 3800, Australia

^2^ Monash Epworth Rehabilitation Research Centre, 185-187 Hoddle Street, Richmond, Victoria 3121, Australia

**Supplementary Materials**

**Supplementary Table S1.** Clinical characteristics of TBI participants.

| **Age (years)** | **Sex** | **Cause**  **of injury** | **PTA (days)** | **GCS (lowest)** | **Time since injury (months)** | **CT/MRI finding** |
| --- | --- | --- | --- | --- | --- | --- |
| 41 | F | Car accident | 19 | 10 | 0.8 | NAD |
| 38 | F | Motorcycle | 7 | 3 | 1 | SAH, SDH |
| 62 | M | Fall of mower | 7 | 14 | 1.2 | Contusion, EDH, SAH |
| 32 | M | Car accident | 22 | 12 | 6 | Contusion, ICH, SAH |
| 48 | M | Motorcycle | 5 | 14 | 3.9 | NAD |
| 45 | M | Bicycle vs. Car | 21 | 8 | 0.7 | SAH |
| 33 | F | Fall of bicycle | 22 | 6 | 1 | SAH |
| 52 | M | Car accident | 14 | 8 | 1.4 | Contusion |
| 21 | F | Pedestrian vs. Car | 3 | 13 | 1.4 | EDH, ICH, SAH |
| 19 | F | Car accident | 63 | 3 | 6.6 | Contusion, EDH, ICH, SAH |
| 22 | M | Car accident | 43 | 15 | 1.8 | SDH |
| 19 | M | Car accident | 24 | 3 | 1.1 | DAI, ICH |
| 19 | M | Car accident | 50 | 3 | 2.7 | DAI, ICH |
| 73 | F | Car accident | 22 | 4 | 2.6 | EDH, SAH, SDH |
| 56 | M | Motorcycle | 23 | 10 | 2.1 | ICH, SAH, SAH |
| 65 | M | Car accident | 24 | 10 | 1.2 | DAI, ICH |
| 32 | F | Car accident | 18 | 11 | 2.3 | ICH |
| 27 | F | Car accident | 7 | 14 | 3.1 | SAH |
| 24 | M | Motorcycle | 39 | 3 | 1.6 | DAI, IVH, SAH |
| 42 | M | Bicycle vs. Truck | 8 | 14 | 1.1 | ICH, SAH, SDH |
| 18 | M | Car accident | 28 | 6 | 1.4 | Contusion, SAH, SDH |
| 18 | M | Pedestrian vs. Bus | 22 | 7 | 1.8 | ICH |
| 62 | M | Motorcycle | Unknown | 15 | 3.4 | SHD |
| 50 | M | Motorcycle | 18 | 13 | 1.2 | ICH |
| 41 | M | Pedestrian vs. Tram | 30 | 6 | 2.5 | ICH |
| 67 | M | Bicycle vs. car | 9 | 8 | 26.93 | DAI, ICH |
| 25 | F | Fall from horse | 34 | 6 | 34.82 | ICH |
| 67 | M | Car accident | 7 | 10 | 19.33 | ICH |
| 42 | M | Car accident | 180 | 4 | 28.34 | DAI, SAH |
| 46 | M | Motorcycle | 15 | 13 | 23.31 | DAI |
| 46 | M | Pedestrian vs. car | 33 | 15 | 13.35 | NAD |
| 50 | M | Motorcycle | 43 | 6 | 26.17 | Contusion, DAI, ICH |
| 62 | M | Car accident | Unknown | Unknown | 28.57 | Pneumocephalus |
| 44 | M | Motorcycle | 34 | 8 | 15.75 | ICH, pneumocephalus, SAH |
| 61 | M | Motorcycle | 23 | 14 | 15.42 | NAD |
| 23 | M | Car accident | 14 | 13 | 17.19 | Contusion, ICH, SAH |
| 20 | F | Pedestrian vs. car | 41 | 3 | 27.58 | Contusion, SAH, SDH |
| 64 | M | Motorcycle | 46 | 9 | 33.70 | DAI, ICH |
| 28 | F | Car accident | 11 | ? | 17.42 | ICH |
| 27 | F | Bicycle vs. tram | 21 | 13 | 21.17 | Contusion, SDH |
| 31 | M | Motorcycle | 24 | 14 | 34.82 | ICH |
| 49 | M | Pedestrian vs. car | 10 | 8 | 16.73 | ICH, SDH |
| 42 | M | Car accident | 18 | 3 | 21.34 | ICH, SAH, SDH |

GSC = Glasgow Coma Scale; PTA = post-traumatic amnesia; NAD = no abnormality detected; SAH = subarachnoid haemorrhage, SHD = subdural haemorrhage, EDH = extradural haematoma; ICH = intracerebral haemorrhage, DAI = diffuse axonal injury. Note: PTA duration were available for *n* = 41patients; acute GCS were available for *n* = 42 patients.

**Supplementary Data – Confidence rating analysis**

To assess whether there were interactions between stimulus category, confidence, and correctness (i.e. correct/incorrect), linear mixed model was used. The outcome measure was the number of observations in each of the stimulus x confidence x correctness interactions. A model was fitted with stimulus category, confidence, correctness, group, and their interaction (stimulus category x confidence x correctness x group) as fixed effects, and participant as a random effect. Overall, there were more correct responses than incorrect, (95% CI, 2.21 – 11.53; *P =* 0.004). The confidence x correctness interaction was significant (95% CI, 10.25 – 23.55; *P* < 0.001), such that there were more correct responses regardless of whether individuals were confident (95% CI, 25.42 – 29.37; *P* < 0.001) or not confident (95% CI, 2.84 – 6.79; *P* < 0.001). There was also a stimulus x confidence interaction (95% CI, 10.25 – 23.55; *P* < 0.001), such that faces were rated less confidently than animals (95% CI, -6.96 - -1.18; *P* = 0.003). However, and more importantly, there were no significant 3- or 4-way interactions (*P* > 0.05); therefore, we felt justified in our decision to forego confidence ratings in our main analysis.

**
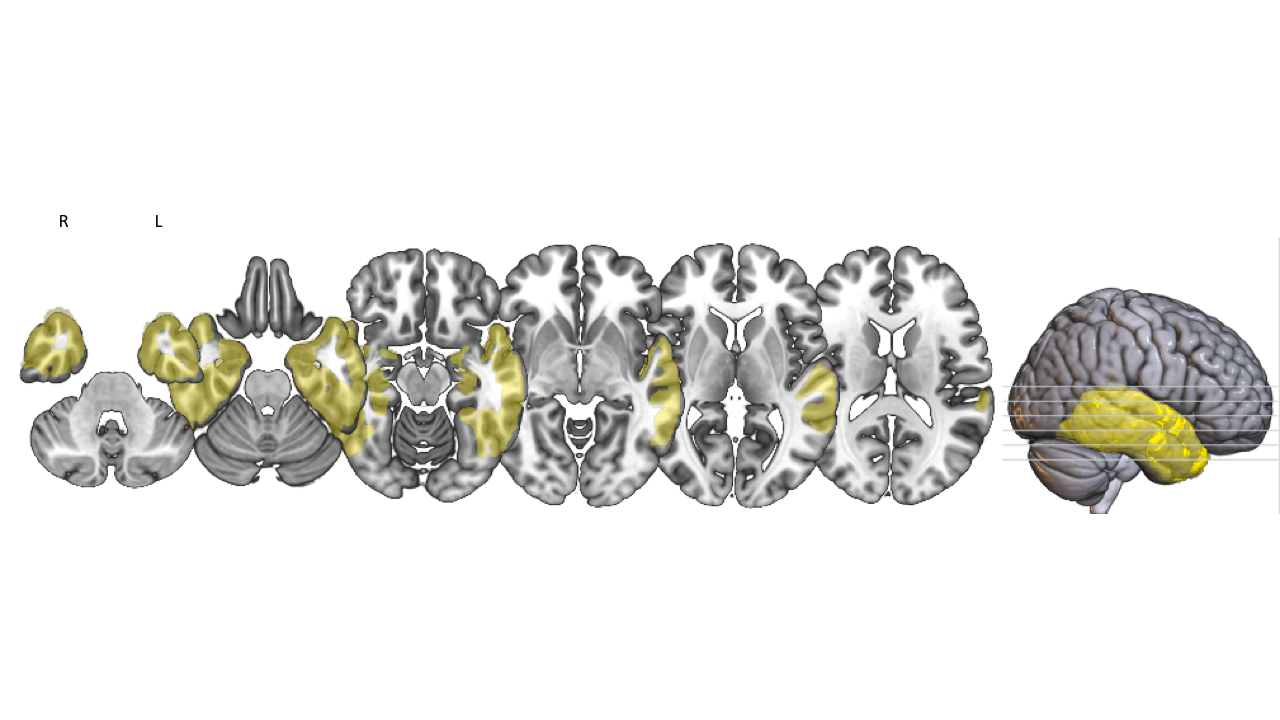
**

**Supplementary Figure S1.** Temporal lobe mask (yellow) used in the group level analysis. The mask was generated from MNI Structural Atlas.

**Supplementary Method – Detailed MRI preprocessing**

Preprocessing of MRI data involved the following: each T1-weighted (T1w) image was corrected for intensity non-uniformity (INU) with N4BiasFieldCorrection ^55^ and skull-stripped with a Nipype implementation of the antsBrainExtraction.sh workflow (using OASIS30ANTs as target template). Brain tissue segmentation of cerebrospinal fluid (CSF), white-matter (WM) and grey-matter (GM) was performed on the brain-extracted T1w using FAST FSL 5.0.9; ^56^. Cortical brain surfaces were reconstructed using recon-all FreeSurfer 6.0.1; ^57^. Volume-based spatial normalisation to two standard spaces (MNI152NLin6Asym, MNI152NLin2009cAsym) was performed through nonlinear registration with antsRegistration (ANTs 2.2.0).

Functional data were skull-stripped using a custom methodology of fMRIPrep. fMRIPrep’s fieldmap-less approach was used to correct for susceptibility distortion using a deformation field resulting from co-registering the BOLD reference to the same-subject T1w-reference with its intensity inverted ^58,59^.

The BOLD reference was then co-registered to the T1w reference using boundary-based registration ^60^ with six degrees of freedom with bbregister (FreeSurfer). Head-motion parameters with respect to the BOLD reference (transformation matrices, and six corresponding rotation and translation parameters) were estimated using FSL’s MCFLIRT ^61^. The BOLD time-series were resampled onto their original, native space by applying a single, composite transform to correct for head-motion and susceptibility distortions. The BOLD time-series were resampled into MNI152NLin6Asym standard space.

Non-steady state volumes were removed from preprocessed BOLD images and spatial smoothing with an isotropic, Gaussian kernel of 6mm FWHM (full-width half-maximum) was applied. Several confounding time-series were calculated based on the preprocessed BOLD: framewise displacement (FD), DVARS and three region-wise global signals. FD and DVARS are calculated, both using their implementations in Nipype ^62^. The three global signals are extracted within the CSF, the WM, and the whole-brain masks. Additionally, a set of physiological regressors were extracted to allow for component-based noise correction CompCor; ^63^. Principal components are estimated after high-pass filtering the preprocessed BOLD time-series (using a discrete cosine filter with 128s cut-off) for the two CompCor variants: temporal (tCompCor) and anatomical (aCompCor). tCompCor components are then calculated from the top 5% variable voxels within a mask covering the subcortical regions. For aCompCor, components are calculated within the intersection of the aforementioned mask and the union of CSF and WM masks calculated in T1w space, after their projection to the native space of each functional run (using the inverse BOLD-to-T1w transformation). Components are also calculated separately within the WM and CSF masks.

Many internal operations of fMRIPrep use Nilearn 0.6.1 ^64^, mostly within the functional processing workflow. For more details of the pipeline, see <https://fmriprep.readthedocs.io/en/1.0.8/workflows.html>.

**Supplementary Table S2.** Regions of significant activation during the episodic encoding task for all participants

| **Contrast** | **Region** |  | **Voxels** | **Peak coordinates** | | | ***P*-value** |
| --- | --- | --- | --- | --- | --- | --- | --- |
|  |  |  |  | X | Y | Z |  |
| Faces | Right fusiform gyrus  Left fusiform gyrus  Right amygdala  Left amygdala  Right fusiform gyrus  Left fusiform gyrus |  | 1111  747  116  110  80  42 | 40  -38  22  -22  32  -32 | -50  -44  -2  -2  -4  -8 | -20  -22  -14  -16  -34  -30 | <0.001  <0.001  <0.001  <0.001  0.002  0.022 |
| Scenes | Right fusiform gyrus  Left fusiform gyrus  Left fusiform gyrus |  | 915  761  34 | 32  -24  -54 | -40  -42  -42 | -12  -16  -10 | <0.001  <0.001  0.044 |
| Animals | Right fusiform gyrus  Left fusiform gyrus  Right fusiform gyrus |  | 1465  1351  101 | 44  -44  30 | -58  -56  -4 | -16  -16  -34 | <0.001  <0.001  <0.001 |

**Supplementary Table S3.** Regions of significant activation during encoding of faces.

| **Contrast** | **Region** |  | **Voxels** | **Peak coordinates** | | | ***P*-value** |
| --- | --- | --- | --- | --- | --- | --- | --- |
|  |  |  |  | X | Y | Z |  |
| HC > TBI | Left middle temporal gyrus |  | 94 | -64 | -52 | 6 | <0.001 |
| TBI | Right fusiform gyrus  Left fusiform gyrus  Right amygdala  Left amygdala  Right fusiform gyrus |  | 1003  686  72  60  54 | 40  -38  22  -22  32 | -50  -46  -4  -2  -4 | -22  -22  -14  -16  -34 | <0.001  <0.001  0.002  0.005  0.008 |
| HC | Right fusiform gyrus  Left fusiform gyrus  Left amygdala  Right amygdala |  | 934  474  50  35 | 36  -42  -18  20 | -74  -54  -6  -4 | -18  -18  -16  -14 | <0.001  <0.001  0.014  0.043 |

**Supplementary Table S4.** Regions of significant activation during encoding of scenes.

| **Contrast** | **Region** | **Voxels** | **Peak coordinates** | | | ***P*-value** |
| --- | --- | --- | --- | --- | --- | --- |
|  |  |  | X | Y | Z |  |
| TBI | Right fusiform gyrus  Left fusiform gyrus | 746  601 | 28  -32 | -42  -42 | -12  -14 | <0.001  <0.001 |
| HC | Right fusiform gyrus  Left fusiform gyrus | 706  526 | 32  -26 | -40  -44 | -14  -16 | <0.001  <0.001 |

**Supplementary Table S5.** Regions of significant activation during encoding of animals.

| **Contrast** | **Region** | **Voxels** | **Peak coordinates** | | | ***P*-value** |
| --- | --- | --- | --- | --- | --- | --- |
|  |  |  | X | Y | Z |  |
| TBI | Right fusiform gyrus  Left fusiform gyrus  Right fusiform gyrus  Left fusiform gyrus | 1331  1244  96  68 | 42  -44  32  -32 | -58  -56  -6  -8 | -20  -16  -34  -30 | <0.001  <0.001  <0.001  0.003 |
| HC | Right fusiform gyrus  Left cerebellum  Right fusiform gyrus | 1271  958  40 | 50  -40  30 | -68  -70  -2 | -12  -18  -34 | <0.001  <0.001  0.035 |

**Supplementary Figure S2.** Signal change and correlation plots for the significant cluster in the left middle temporal gyrus extending to the superior temporal sulcus during face processing. A) Plot of the COPE values extracted from the significant cluster. The difference in COPE values between the TBI group and healthy controls was significant (*P =* 0.003). B) Overall, there was no direct relationship between COPE values and the overall dprime scores for face stimuli (*P =* 0.167). C) There was a negative weak correlation that trended towards significant when considering the first recognition run only (*P* = 0.088). D) No significant association was apparent when considering the second recognition run only (*P =* 0.527).
